# Chylothorax as an Unusual Initial Manifestation of Diffuse Large B-Cell Lymphoma in an 86-Year-Old Woman: A Reflection of Diagnostic Reasoning and Age in Therapeutic Decisions

**DOI:** 10.7759/cureus.102494

**Published:** 2026-01-28

**Authors:** Diogo Dias Ramos, Amanda Hirschfeld, Inês Fiúza M. Rua, André Valente, Ana M Serrano

**Affiliations:** 1 Internal Medicine, Unidade Local de Saúde de São José, Lisbon, PRT; 2 Oncology, Instituto Português de Oncologia de Lisboa Francisco Gentil (IPO Lisboa), Lisbon, PRT

**Keywords:** dyspnea, large b-cell diffuse lymphoma, malignant chylothorax, non-hodgkin's lymphoma, pleural effusions

## Abstract

Chylothorax is a rare presentation of lymphoproliferative disorders, responsible for only a small proportion of non-traumatic pleural effusions in large series. In older adults, its recognition can be challenging, often delaying diagnosis and treatment. We report an 86-year-old woman admitted with progressive dyspnea and a large right pleural effusion. Thoracentesis yielded a milky fluid with high triglycerides, confirming chylothorax. Cytology was negative for malignant cells, and a CT scan revealed an extensive retroperitoneal mass encasing the aorta and inferior vena cava, highly suggestive of lymphoproliferative disease. An inguinal lymph node biopsy established the diagnosis of diffuse large B-cell lymphoma. The patient was managed with a low-fat diet enriched with medium-chain triglycerides, subcutaneous octreotide, and chest drainage, leading to complete resolution of the effusion. Corticosteroid therapy was initiated, resulting in clinical improvement, weight gain and marked recovery of performance status, leading to the discharge of the patient. She was subsequently referred to Haematology and proposed for chemotherapy. Despite advanced age, the patient achieved significant improvement, reinforcing that chronological age alone should not preclude adequate therapy and even in very old patients, targeted treatment can yield meaningful recovery and quality of life.

## Introduction

Chylothorax is defined as the accumulation of lymphatic fluid within the pleural space due to disruption or obstruction of the thoracic duct. While trauma and postoperative injury are classical causes, malignancy accounts for approximately one-third of cases, with lymphoma, particularly non-Hodgkin subtypes, as the leading aetiology [1,2].

However, presentation with chylothorax as the first and sole manifestation of lymphoma remains rare, especially in very elderly patients. Such cases pose diagnostic and ethical challenges: the rarity of the presentation often delays tissue diagnosis, and therapeutic decisions may be influenced by chronological age rather than biological fitness. Early recognition is critical, as delayed diagnosis may lead to significant respiratory morbidity, nutritional compromise, and infectious complications, while in aggressive lymphomas such as diffuse large B-cell lymphoma (DLBCL), timely treatment is a major determinant of prognosis, particularly in older adults.

We present a case of DLBCL initially manifesting as chylothorax in an 86-year-old woman, which illustrates the need for comprehensive evaluation and individualised management in older adults.

## Case presentation

An 86-year-old woman presented to the emergency department with progressive dyspnea and lower-limb oedema. Her relevant past medical history included a history of hypertension, dyslipidaemia, untreated type 2 diabetes mellitus, depressive disorder, suspected major neurocognitive disorder with behavioural disturbances, and severe diabetic retinopathy with significant visual impairment. She also had a history of pulmonary embolism (2009) and right femoral neck fracture treated with hip prosthesis (2014). Her chronic medication included duloxetine, perindopril, acenocoumarol, simvastatin, alprazolam, and zolpidem.

In the emergency department, she was conscious and cooperative, eupneic on oxygen therapy (6 L/min via face mask), with oxygen saturation of 90-92%, blood pressure of 119/54 mmHg, heart rate of 89 bpm, and afebrile. Pulmonary auscultation revealed absent breath sounds over the lower half of the right hemithorax and reduced sounds at the left base. Peripheral oedema grade 2+/3+ with bilateral pitting was present, and abdominal examination was unremarkable.

Initial laboratory evaluation showed mild leukocytosis with neutrophilia, an elevated C-reactive protein, mild renal impairment with a slight hyponatremia, and a cholestatic pattern of liver enzyme elevation (Table 1).

**Table 1:** Initial laboratory evaluation

Blood Work	Patient Results	Normal Range
Haemoglobin	13.5 g/dL	12.0 – 15.0
Hematocrit	41.60%	35 – 46
Mean Corpuscular Volume (MCV)	87.9 fL	78.0 – 96.0
Mean Corpuscular Haemoglobin (MCH)	28.5 pg	26.0 – 33.0
White Blood Cells (WBC)	12.09 ×10⁹/L	4.5 – 11.0
Neutrophils (Absolute Count)	10.65 ×10⁹/L	2.0 – 8.5
Lymphocytes (Absolute Count)	0.78 ×10⁹/L	0.9 – 3.5
Monocytes (Absolute Count)	0.45 ×10⁹/L	0.2 – 1.0
Eosinophils (Absolute Count)	0.17 ×10⁹/L	0.0 – 0.6
Basophils (Absolute Count)	0.04 ×10⁹/L	0.0 – 0.1
Platelets	194 ×10⁹/L	150 – 450
Prothrombin Time (PT)	11.4 s	9.4 – 12.5
INR (International Normalized Ratio)	0.95	0.80 – 1.20
aPTT (Activated Partial Thromboplastin Time)	24.5 s	25.1 – 36.5
Urea	54 mg/dL	21 – 43
Creatinine	0.78 mg/dL	0.57 – 1.11
Estimated GFR (CKD-EPI)	69 mL/min/1.73 m²	≥ 90
Sodium	131 mEq/L	136 – 145
Potassium	4.0 mEq/L	3.5 – 5.1
Calcium	8.5 mg/dL	8.40 – 10.20
Uric Acid	4.1 mg/dL	2.60 – 6.00
Total Bilirubin	0.73 mg/dL	0.20 – 1.20
AST (Aspartate Aminotransferase)	15 U/L	5 – 34
ALT (Alanine Aminotransferase)	11 U/L	0 – 55
GGT (Gamma-Glutamyl Transferase)	212 U/L	9 – 36
Alkaline Phosphatase	239 U/L	40 – 150
LDH (Lactate Dehydrogenase)	244 U/L	125 – 220
C-reactive Protein (CRP)	36.4 mg/L	< 5.0 mg/L

Urinalysis revealed glycosuria (200mg/dL), mild proteinuria (50mg/dL), and leukocyturia (1687/µL) with no other findings.

**Figure 1:**
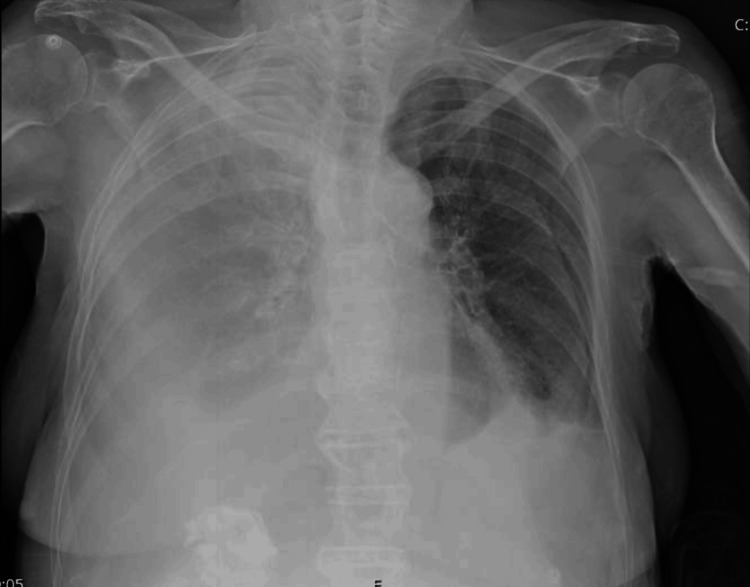
Chest X-ray showing right-sided opacification and small left-sided pleural effusion

Chest X-ray showed near-complete right-sided opacification suggestive of massive right pleural effusion and a small left-sided pleural effusion (Figure 1). 

A presumed diagnosis of acute decompensated heart failure secondary to respiratory infection was made, and the patient started empirical antibiotic therapy with amoxicillin-clavulanate (1,200 mg three times daily) and intravenous furosemide.

Thoracentesis was performed for symptomatic relief and diagnostic purposes, evacuating 1,300 mL of turbid, milky fluid. Biochemical analysis confirmed chylothorax (pH 7.5, protein 44.6 g/L, triglycerides 364 mg/dL, cholesterol 87 mg/dL), which was inconsistent with the initial working diagnosis of acute decompensated heart failure. These findings, together with the right-sided predominance of the effusion, were anatomically consistent with disruption or obstruction of the thoracic duct along its intrathoracic course. Cytology showed reactive mesothelial cells without malignancy and microbiological cultures were negative (Table 2).

**Table 2:** Pleural fluid biochemical and microbiological analysis

Pleural Fluid Analysis	Patient Results	Normal Range
Macroscopic Appearance	Turbid, Light Yellow	Clear, Straw-Colored
pH (Pleural Fluid)	7.5	~7.60
Leukocytes (Pleural Fluid)	801 /µL	< 1000 (Transudate)
Polymorphonuclear Cells	63 /µL	—
Mononuclear Cells	738 /µL	—
Erythrocytes	5.0 ×10³ /µL	—
Total Proteins (Pleural Fluid)	44.6 g/L	< 30
Albumin (Pleural Fluid)	24.8 g/L	—
Glucose (Pleural Fluid)	291 mg/dL	> 60
LDH (Pleural Fluid)	142 U/L	< 200
Triglycerides (Pleural Fluid)	364 mg/dL	< 50
Total Cholesterol (Pleural Fluid)	87 mg/dL	< 60
Amylase (Pleural Fluid)	9 U/L	< 1.5 × Serum Value
Total Bilirubin (Pleural Fluid)	1.0 mg/dL	Pleural/Serum Ratio < 0.6
Adenosine Deaminase (ADA)	15.9 U/L	< 31
Microbiological Cultures (Aerobic)	No Growth after Five Days	Sterile
Microbiological Cultures (Anaerobic)	No Growth after Five Days	Sterile

Due to an iatrogenic pneumothorax, a chest drain (28 Fr) was inserted, and the patient was started on a low-fat diet enriched with medium-chain triglycerides and subcutaneous octreotide 100µg t.i.d. Daily drainage progressively decreased, allowing removal of the drain after eight days with radiographic resolution of the effusion.

As part of the etiological investigation of the chylothorax, a CT thoraco-abdomino-pelvic imaging was ordered, revealing a 13 × 10 × 7 cm retroperitoneal mass surrounding the aorta and inferior vena cava and extending to the mesenteric root, compatible with a large conglomerate of lymph nodes. Beta-2-microglobulin was also elevated (3.2 mg/L; reference range: 0.97 - 2.64mg/L). 

An ultrasound-guided inguinal lymph node biopsy was performed confirming the diagnosis of DLBCL. The hospital course was further complicated by a transient nosocomial pneumonia and candiduria, both successfully treated.

Given her preserved functional status and improvement, she was started on oral prednisolone 20 mg daily, with excellent tolerance, increased appetite, and weight gain of 4.5 pounds. At follow-up, she was clinically stable, functionally independent (ECOG performance status of 0), and was referred to Haematology, where she was proposed for chemotherapy.

## Discussion

Chylothorax secondary to malignancy is most often associated with lymphomas, particularly non-Hodgkin subtypes such as DLBCL [1-3]. Obstruction or infiltration of the thoracic duct leads to lymphatic leakage into the pleural cavity, producing a milky exudate rich in triglycerides. The right-sided predominance observed in this patient suggests ductal involvement below the fifth thoracic vertebra [4].

Chylothorax remains an uncommon initial manifestation of lymphoma, accounting for less than 5% of non-traumatic cases in large series [1,2].

The conservative strategy combining dietary fat restriction with medium-chain triglycerides, octreotide, and pleural drainage remains first-line therapy [3,4]. This approach aims to reduce chyle flow, promote healing of the duct and allow spontaneous resolution, as observed here. Surgical or interventional management is reserved for persistent high-output chylothorax.

In older adults, diagnosis and treatment are often constrained by perceptions of futility. However, evidence from large registries demonstrates that even patients aged ≥85 years with DLBCL can benefit from therapy with curative intent when functionally fit [5]. Few case reports describe chylothorax as the presenting feature of lymphoma in the very elderly, but similar outcomes have been documented when early recognition and multidisciplinary coordination occur [6].

## Conclusions

This case highlights chylothorax as a rare initial manifestation of DLBCL in a very elderly patient, emphasising the diagnostic challenges associated with non-traumatic chylothorax and negative pleural fluid cytology. In this context, a high index of suspicion and a structured diagnostic approach are critical for timely identification of the underlying lymphoproliferative disorder. Conservative management, including dietary modification, octreotide, pleural drainage, and corticosteroid therapy, resulted in complete resolution of the effusion and marked functional improvement.

Finally, advanced age should not, in isolation, limit therapeutic decisions. Careful assessment of functional status and biological fitness, supported by a multidisciplinary and patient-centred approach, can allow meaningful recovery and access to disease-directed treatment even in very old patients.
